# Can the Liebowitz Social Anxiety Scale - Self-Report Version Be Used to Differentiate Clinical and Non-Clinical SAD Groups among Brazilians?

**DOI:** 10.1371/journal.pone.0121437

**Published:** 2015-03-26

**Authors:** Larissa F. Santos, Sonia R. Loureiro, José A. S. Crippa, Flávia L. Osório

**Affiliations:** 1 Department of Neurosciences and Behavioral Sciences, Faculty of Medicine of Ribeirão Preto—USP, Ribeirão Preto, São Paulo, Brazil; 2 National Institute of Technology and Translational Medicine—CNPq, Brazil; West China Hospital of Sichuan University, CHINA

## Abstract

**Background:**

The Liebowitz Social Anxiety Scale (LSAS) was the first evaluation instrument developed for screening for the signs and symptoms of Social Anxiety Disorder (SAD) and is currently still the most used worldwide. The aim of this study is to evaluate the ability of the LSAS - self-report version (LSAS-SR) to discriminate different Social Anxiety Disorder (SAD) clinical groups.

**Method:**

The sample was composed of Brazilians university students, allocated into three different groups, i.e., cases (C=118), non-cases (NC=95) and subclinical cases (SC=39). To achieve the aim, calculations of the ROC Curve and ANOVA were performed.

**Results:**

The results found were excellent regardless of the technique used, highlighting the discriminatory capacity of the LSAS-SR. The score equal to or greater than 32 is suggested as a cutoff score for the Brazilian population, since this presented balance between the standards evaluated and the ability to differentiate both clinical and subclinical SAD cases from non-cases.

**Conclusion:**

Despite the specific sample used in this study being composed only of university students, the use of the LSAS-SR can be indicated, in the Brazilian setting, for SAD screening in both clinical and research contexts.

## Introduction

Social Anxiety Disorder (SAD) is characterized by a strong and persistent fear of social situations which could cause embarrassment. In many cases, individuals who are affected by SAD avoid these kinds of situations, which is related to social impairment. When avoidance is not possible, physiological symptoms, such as sweating and flushing, can be seen. It is important to note that these people are able to realize that the fear experienced in this situation is irrational [1].

According to the fourth edition of the Diagnostic and Statistical Manual of Mental Disorders (DSM-IV), SAD can be classified as generalized when the individual feels fear in almost all social situations, for example, speaking in public, going to parties, or participating in small groups. When the discomfort is only generated in certain situations, it can be classified as circumscribed [1].

When a number of SAD symptoms are present without clear social impairment, these cases are classified as subclinical SAD [2].

The “gold-standard” for psychiatry diagnoses are structured clinical interviews, especially the Structured Clinical Interview for the DSM-IV (SCID-IV). These assessment scales can be important in screening for possible cases and can help in making the clinical diagnoses.

Considering Social Anxiety Disorder (SAD), the number of evaluation instruments has grown considerably over the last decade. According to a review study [3] there are approximately 25 instruments for evaluating the disorder, with the majority having been studied from the psychometric point of view and having shown good indicators.

Among them, the Liebowitz Social Anxiety Scale (LSAS) was the first instrument developed specifically for the evaluation of SAD, in 1987, by Michael Liebowitz [4], in the United States. It was proposed as a clinician-administered scale consisting of 24 items, on two subscales, which evaluate the symptoms of fear and the avoidance of social situations experienced by the individual in the previous week. The score is obtained through a Likert scale from 0 to 3 (none/never to severe/usually). In this same period, the self-report version of the scale (LSAS-SR) was proposed by Cox, Ross, Swinson and Derenfeld [5].

The first psychometric study using the LSAS was only conducted 10 years after its development [6] and the results showed adequate internal consistency (α = 0.68 to 0.98), moderate to excellent convergent validity (Social Interaction Anxiety Scale = 0.47 to 0.76; Social Phobia Scale = 0.50 to 0.77), excellent divergent validity (Hamilton Anxiety Scale = 0.48; Beck Depression Inventory = 0.39; Hamilton Depression Rating Scale = 0.52) and excellent predictive validity (0.58 to 0.67).

Specifically regarding the discriminant validity, ten studies were found [6–15] which indicated that SAD participants presented significantly higher scores than participants without SAD or with other anxiety disorders, regarding both subscales and the overall score.

The cutoff points present many differences according to the culture of the sample, for example, the studies conduced in the USA [11,13] both used a cutoff score of 30 points. However, in Europe, the cutoff scores were different in each country, a Turkish study suggested that the score should be higher than 50 [14], while a Spanish study suggested a score between 19.6 and 26.1[7].

Subsequent studies [16,17], consisting of large and diverse samples and using refined methodology, have considered the self-report version valid and reliable, since the psychometric properties presented by the SR version were as satisfactory as the clinician-administered format, with the advantage of its application being easier and faster [12]. Currently, the LSAS is the instrument most used worldwide for the evaluation of signs and symptoms of SAD, especially in clinical studies [18,19]. However, it should be noted that the LSAS-SR is an instrument that evaluates only two of the diagnostic criteria of SAD, the fear and avoidance associated with the disorder, without including its other diagnostic criteria, e.g., the physiological symptoms (Criteria B, DSM-IV), and the impairments related to the SAD symptoms (Criteria E, DSM-IV). Accordingly, it can be asked whether the scale is capable of discriminating SAD sufferers, being restricted to the aspects evaluated, considering that the experience of impairments or the interference of the symptoms in people’s everyday lives is the differentiating sign between clinical and subclinical cases. A further question would be whether the amount/intensity of the experiences of fear and avoidance is a clinical indicator of the severity.

To answer these questions, the aim of this study was to examine the discriminant validity and find the LSAS-SR cutoffs for the Brazilian population, with a clinical sample of SAD cases, non-cases and subclinical cases.

## Method

### Participants

The sample was composed of university students, of both genders, aged between 18 and 35 years. They were included only after their agreement and signing of the terms of informed consent.

Module F of the Structured Clinical Interview for the DSM-IV (SCID-IV) was used for the inclusion of the participants in each clinical group, conforming to the criteria described in Table 1. The SCID-IV was administered by trained researchers with previous experience in psychiatric assessment.

**Table 1:** Criteria for inclusion of the participants into Social Anxiety Disorder groups, according to the diagnoses.

Group	Criteria	N
Case (C)	SCID-IV—Module F: positive	118
Non-Case (NC)	SCID-IV—Module F: negative	95
Subclinical (SC)	SCID-IV: positive, except for the criterion E—social impairments	39

Note. Mini-SPIN = Social Phobia Inventory—short version; SCID-IV = Structured Interview for the DSM-IV; N = Number of participants

The exclusion criteria, for all the subgroups, were the use of neuroleptics (N = 0) or the presence of the following psychiatric comorbidities (N = 23): psychotic manifestations, current depression, recurrent depression, current eating disorder, obsessive compulsive disorder, hypomanic/manic episodes or panic disorder, as well as the incorrect completion of the instruments (N = 41), a further 151 subjects were not located or were not interested in continuing to participate.

### Measures

#### Liebowitz Social Anxiety Scale, self-report version (LSAS-SR; Liebowitz, 1987)[4]

The scale is composed of 24 items, scored on a Likert-type scale of four points, on two subscales related to the fear and the avoidance of different social situations experienced in the previous week. Its total score varies from 0 to 144 points and it has been adapted into Brazilian Portuguese, with excellent psychometric properties (internal consistency: α = 0.90–0.96; test-retest reliability: Intraclass Correlation Coefficient = 0.81 and Pearson’s = 0.82; convergent validity: r = 0.21 to 0.84) [20].

#### Mini-Social Phobia Inventory (Mini-SPIN; Connor, Kobak, Churchill, Katzelnick, & Davidson, 2001)[21]

This reduced instrument consists of three of the 17 items of the SPIN (items 6, 9, 15), which proved, in the psychometric study, to be the most discriminative for participants with SAD. It was translated and adapted into Brazilian Portuguese (internal consistency: α = 0.49 to 0.73; sensitivity: 0.94; specificity: 0.46) [22,23].

#### Structured Clinical Interview for the DSM-IV—Diagnostic and Statistical Manual of Mental Disorders, 4th ed. (SCID-IV; First, Spitzer, Gibon, & Williams, 1997)[24]

This instrument consists of an interview script, composed of ten modules, used for the development of psychiatric clinical diagnoses based on the DSM-IV. It should be noted that Module F refers to Social Anxiety and has been translated and adapted into Portuguese [25].

#### Identification Questionnaire

This questionnaire is composed of 16 items aimed at the sociodemographic characterization of the participants.

### Procedure

Consent was obtained for the performance of the study from the Human Research Ethics Committee of the Clinical Hospital of Ribeirao Preto Medical School, University of São Paulo (CEP Process: 11570/2003-HCRP).

For the data collection, permission was sought from the coordinators of the Universities. Following their approval, courses and disciplines with the highest number of students enrolled were chosen by convenience, and then the professor was asked for authorization to perform the data collection in the classroom. At this stage the study aims and the voluntary nature of their participation in the study were explained to the students. Those who agreed to participate were given the terms of informed consent and after their formal acceptance were provided with an application notebook containing the Mini-SPIN and Identification Questionnaire instruments.

Primarily, in order to perform an initial screening for potential cases of SAD, a total of 2614 participants were evaluated in the classroom, where they responded to the Mini-SPIN. Of these, 201 chose not to continue, citing lack of interest or availability, 53 were excluded for being aged below 17 or above 35 years, 41 for incorrect completion of the instruments and five due to the use of neuroleptics, giving a final sample of 2314 participants.

Next, in order to confirm the SAD diagnosis and the classification of its subtypes, those participants with positive scores for SAD according to the Mini-SPIN were contacted by telephone to respond to module F of the SCID-IV (N = 473). A control group of 253 participants, with negative Mini-SPIN scores, was also interviewed by telephone in order to confirm the absence of this diagnosis. It was not possible to make contact with 217 of these and 83 claimed to have no more interest in participating. According to the SCID-IV inclusion criteria, 178 participants were classified as SAD cases, 194 as non-cases and 54 as subclinical cases, from a total of 426 participants.

Finally, these participants were evaluated in person, individually, by applying the LSAS-SR and the complete SCID-IV and allocated into the clinical groups following the previously described parameters. After applying the exclusion criteria, the final sample was composed of 252 participants.

Data were coded and entered manually into a database and the statistical program SPSS version 13.0 [26] was used for the analyses. The demographic and clinical data of the sample were analyzed using descriptive and parametric statistical tests. To compare the groups, according to sociodemographic and clinical data, the chi-square test and ANOVA were used. These tests were chosen due to the sample presenting a parametric distribution.

For the study regarding the discriminative validity of the LSAS, the ROC curve (*Receiver Operating Characteristic Analyses*) was calculated, with the aim of analyzing the cutoff points that favor discrimination between the participants classified as clinical cases, non-cases and subclinical cases of SAD. The Sensitivity (S), Specificity (E), Positive Predictive Value (PPV), Negative Predictive Value (NPV) and Misclassification Error Rate (MER) [27] were also calculated. The Student t-test and ANOVA were used to compare the variables according to the gender of the participants, the clinical groups and the SAD subtypes, using the Bonferroni post-hoc test. The significance level adopted was p≤0.05.

## Results

### Characterization of the sample

The sociodemographic characterization of the sample, according to the clinical groups, is shown in Table 2.

**Table 2:** Sociodemographic characterization of the sample (N = 252).

Variable	C (N = 118) N (%)	NC (N = 95) N (%)	SC (N = 39) N (%)	Statistic
**Gender**				
Female	81(68.6)	56 (58.9)	30 (76.9)	χ^2^ = 4.789
Male	37 (31.4)	39 (41.1)	9 (23.1)	p = 0.91
**Mean age (SD)**	22,63 (5.4)*	20.93 (2.86)*	21.29 (3.1)	F = 4.150 p = 0.017*
**Field of study**				
Exact	37 (31.4)	33 (34.7)	13 (33.3)	χ^2^ = 0.327
Humanities	14 (11.9)	11 (11.6)	5 (12.8)	p = 0.988
Biological	67 (56.8)	51 (53.7)	21 (53.9)	
**Year of course**				
1^st^ and 2^nd^	75 (63.6)	70 (73.7)	30 (76.9)	χ^2^ = 3.759
3^rd^ and 4^th^	43 (36.4)	25 (26.3)	9 (23.1)	p = 0.153

Note. C = Cases; NC = Non-Cases; SC = Subclinical cases; N = Frequency; % = Percentage; SD = Standard Deviation; χ^2^ = Chi-squared test; F = ANOVA;

* = Statistically significant difference.

The final sample, composed of 252 participants, presented a predominance of female students, from a public university, enrolled in the first years of the university course, mainly in the biological sciences area, independent of the clinical group. Overall, the sample showed no statistically significant differences regarding the sociodemographic data. This was seen only in relation to the age variable, where a small difference was found between the C and NC groups, with a higher mean age in the C group compared to the NC group.

**Fig 1:**
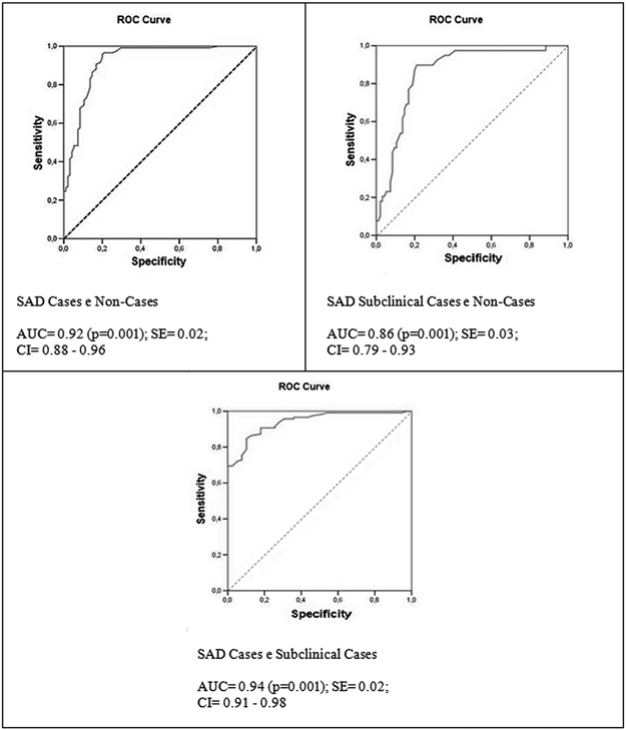
Area under the ROC curve of the total score of LSAS-SR for a sample of SAD cases (N = 118) and non-cases (N = 95); subclinical cases (N = 39) and non-cases (N = 95); and between cases (N = 118) and subclinical cases (N = 39). SAD = Social Anxiety disorder; AUC = Area under the curve; SE = standard error; CI = confidence interval.

Analysis was performed, using the ROC curve, between the following clinical groups: a) cases and non-cases, b) subclinical cases and non-cases, and c) cases and subclinical cases, the results of which are presented in Fig. 1.

In the three cases, excellent areas under the curves were found, being greater than 0.90 between the C and NC groups and between the C and SC groups and slightly lower (0.86) between the SC and NC groups.

Regarding the cutoff scores, those which presented the best balance between the evaluated parameters were similar between the C and NC groups and between the SC and NC groups, as can be seen in Table 3.

**Table 3:** Relative values for the study of discriminative validity of the LSAS-SR in a sample of SAD cases (N = 118) and non-cases (N = 95), and a sample of SAD subclinical cases (N = 39) and non-cases (N = 95).

Score	C and NC					SC and NC				
	S	E	PPV	NPV	MER	S	E	PPV	NPV	MER
26	0.99	0.70	0.80	0.98	0.07	0.90	0.70	0.55	0.94	0.12
27	0.98	0.71	0.81	0.97	0.07	0.90	0.71	0.56	0.94	0.11
30	0.96	0.76	0.83	0.94	0.06	***0.90***	***0.76***	***0.61***	***0.95***	***0.10***
31	0.96	0.77	0.84	0.96	0.06	***0.90***	***0.77***	***0.62***	***0.95***	***0.09***
32	***0.96***	***0.79***	***0.85***	***0.95***	***0.06***	***0.90***	***0.79***	***0.64***	***0.95***	***0.09***
33	***0.95***	***0.80***	***0.85***	***0.93***	***0.06***	***0.87***	***0.80***	***0.64***	***0.94***	***0.09***
34	***0.96***	***0.80***	***0.85***	***0.94***	***0.06***	***0.87***	***0.80***	***0.64***	***0.94***	***0.09***
35	***0.94***	***0.80***	***0.85***	***0.91***	***0.06***	***0.87***	***0.80***	***0.65***	***0.95***	***0.09***
36	***0.91***	***0.81***	***0.86***	***0.88***	***0.06***	0.79	0.81	0.63	0.90	0.10
37	***0.91***	***0.81***	***0.84***	***0.88***	***0.06***	0.79	0.81	0.63	0.90	0.10
38	***0.91***	***0.83***	***0.87***	***0.88***	***0.06***	0.77	0.83	0.65	0.90	0.09
39	***0.91***	***0.83***	***0.87***	***0.88***	***0.06***	0.74	0.83	0.64	0.89	0.11
45	0.83	0.86	0.88	0.80	0.08	0.61	0.86	0.65	0.84	0.10
50	0.72	0.89	0.89	0.72	0.10	0.49	0.89	0.65	0.81	0.11

Note. C = Cases; NC = Non-Cases; SC = Subclinical cases; S = Sensitivity; E = Specificity; PPV = Positive Predictive Value; NPV = Negative Predictive Value; MER = Misclassification Error Rate.

According to Table 3, considering the C and NC groups, it can be observed that the interval between scores 32 and 39 presented adequate balance between the evaluated parameters, with sensitivity greater than 0.91 and specificity greater than 0.79. For the discrimination between the SC and NC groups the interval between scores 30 and 35 showed the most acceptable balance, with sensitivity values greater than 0.87 and specificity greater than 0.76.

Table 4 shows the S, E, PPV, NPV and MER values for the discrimination between the C and SC groups.

**Table 4:** Relative values for the study of discriminative validity of the LSAS-SR in a sample of SAD cases and subclinical cases.

Score	S	E	PPV	NPV	MER
26	0.99	0.46	0.85	0.95	0.14
27	0.98	0.49	0.85	0.90	0.14
39	***0.91***	***0.82***	***0.94***	***0.74***	***0.11***
40	***0.88***	***0.82***	***0.94***	***0.69***	***0.13***
41	***0.88***	***0.82***	***0.94***	***0.69***	***0.13***
42	***0.87***	***0.82***	***0.94***	***0.68***	***0.14***
43	***0.86***	***0.87***	***0.95***	***0.68***	***0.13***
44	***0.85***	***0.90***	***0.96***	***0.66***	***0.14***
45	***0.83***	***0.90***	***0.96***	***0.64***	***0.15***
50	0.72	0.95	0.97	0.53	0.22
51	0.69	0.97	0.99	0.51	0.23

Note. S = Sensitivity; E = Specificity; PPV = Positive Predictive Value; NPV = Negative Predictive Value; MER = Misclassification Error Rate.

To discriminate between the SAD case participants and those with subclinical SAD, the interval between scores 39 and 45 was indicated, since this presented a satisfactory balance between the parameters analyzed, with sensitivity and specificity both above 0.82. The capacity of the LSAS-SR to discriminate between the different participants according to the variables gender, sample group and subtype of the disorder (SAD generalized or circumscribed) was also verified, with the results presented in Table 5.

**Table 5:** Mean score of the LSAS-SR and its subscales according to gender and sample group.

	Total Mean (SD)	FS Mean (SD)	AS Mean (SD)
**Gender**			
Fe (N = 167)	54.38 (32.04)	28.95 (16.49)	25.43 (16.19)
M (N = 85)	44.53 (30.63)	23.30 (15.62)	21.24 (15.63)
Statistic	t = 2.35 (p≤ 0.02*)	t = 2.63(p≤ 0.009*)	t = 1.97 (p≤ 0.05*)
AUC	0.59 (p = 0.04)	0.64 (p = 0.01)	0.64 (p = 0.009)
**Group**			
C (N = 118)	70.91 (27.20)	37.20 (13.63)	33.70 (14.34)
NC (N = 95)	24.81 (19.80)	13.41 (10.75)	11.40 (9.88)
SC (N = 39)	54.90 (22.70)	29.49 (11.46)	25.41 (12.32)
Statistic	F = 97.91 (p≤ 0.001*)	F = 99.63 (p≤ 0.001*)	F = 83.84 (p≤ 0.001*)
**Subtype**			
G (N = 56)	77.08	40.11	36.97
C (N = 62)	64.07	33.98	30.09
Statistic	t = -2.66 (p≤ 0.009*)	t = -2.49 (p≤ 0.014*)	t = -2.67 (p≤ 0.009*)
AUC	0.54 (p = 0.05)	0.60(p = 0.008)	0.58(p = 0.04)

Note. SD = Standard deviation; FS = Fear Subscale; AS = Avoidance subscale; Fe = Female; M = Male; GP = General Population; C = Case; NC = Non-Case; SC = Subclinical Case; G = Generalized; C = Circumscribed; t = t test; F = ANOVA;

* = significant difference; AUC: Area under the ROC curve.

In all the analyzes performed, statistically significant differences (p ≤ 0.001) were found, either for the total scale or for the subscales, considering the t-test, ANOVA or the area under the ROC curve. It is noteworthy that, in relation to gender, the mean score was higher among the female participants. Among the sample groups, according to the post-hoc analysis (Bonferroni), the C group had the highest mean score, followed by the SC and NC groups. Considering the diagnosis subtype, generalized or circumscribed SAD, the mean score was higher among the participants with the generalized disorder, in the three situations analyzed.

## Discussion

Considering the area encountered under the ROC curve, this was excellent in all three analyzes, being slightly lower between the SC and NC groups. In relation to the definition of the cutoff scores, the intervals between 32 and 39 for the discrimination between C and NC, and 30 to 35 for SC and NC, were those with the best balance of the evaluated parameters. Thus, considering the groups together, a score equal to 32 for use in the context of SAD screening is suggested, as this allowed a refined differentiation of both clinical and sub-clinical cases from the non-cases.

It is interesting to highlight that the LSAS-SR, in addition to being sensitive for the discrimination of the participants of the C and NC groups, was able to differentiate the C group from the SC group, while not directly addressing the issue of functional impairments, a key factor for this classification [2]. Therefore, the score with the best balance between sensitivity and specificity was equal to 44, however, the interval between the scores 39 and 44 also presented adequate standards for this evaluation, with a sensitive variation between these.

Previous studies differ in relation to the best cutoff to be used, possibly due to the samples and the cultural contexts in which the studies were performed. For example, in another study conducted in the Brazilian context, using a very specific sample which consisted of only patients with Parkinson’s disease [9], a cutoff score between 41 and 42 was suggested, since this interval favored the best balance between sensitivity and specificity.

In two studies conducted in the United States [11,13], with clinical populations of cases and non-cases, both arrived at the same cutoff value, suggesting a score of 30, for the total scale, a value close to that of the present study. Furthermore, European studies have presented a great difference in relation to the cutoff score suggested, perhaps because they were conducted in very different countries from the cultural point of view, such as Spain [7] and Turkey [14]. In the Spanish study [7] a cutoff score of between 19.6 and 26.1 was suggested, while, the Turkish study [14] indicated that the score should be higher than 50. A possible explanation for the differences in the cutoff results in these studies is the cultural specificities of the context of each study, as these specificities tend to influence the diagnosis and the recognition of certain individual characteristics as SAD symptoms.

It should also be highlighted that the scale was able to discriminate the groups according to the variables, these being, gender, sample group and subtype of the disorder. The higher score among the women corroborates data from the literature, since female participants tend to present a higher incidence of symptoms than male participants, indicating a higher prevalence and severity of the disorder among women [28].

Regarding the discrimination between the clinical groups, the group C participants presented a mean score significantly higher, in both the total score and in the scores of the subscales, than the SC group, with both groups presenting higher means than the NC group. This indicates that the symptomatological groups present a higher prevalence regarding SAD related fear and avoidance, as expected. Similarly, those individuals who had been diagnosed with generalized SAD, which is characterized by the presence of fear and avoidance in various social situations, presented higher scores than those patients with circumscribed SAD, who show symptoms of the disorder in specific situations, reinforcing previously encountered data [29].

The results evidence the cultural issues surrounding the diagnosis of SAD, since the constructs shyness and embarrassment, closely linked to the disorder, can be evaluated very differently in relation to the context in which they operate. For example, in certain cultures, they are viewed as positive aspects because the individual is considered introspective, a highly valued characteristic, especially in the Eastern cultures [30].

Based on the data discussed, it can be stated that although the instrument evaluates only two of the SAD diagnostic criteria according to the DSM-IV (*A: marked and persistent fear of one or more social or performance situations; D: The feared social or performance situations are avoided)*, it is able to correctly identify the disorder sufferers, revealing its discriminative validity in the Brazilian context.

In general, studies on the discriminant validity of SAD screening/diagnosis instruments are of fundamental importance so that possible sufferers of the disorder can be correctly identified, both in order to offer them appropriate treatment, as well as for the refinement of clinical research methodologies.

The main limitation of this study relates to the sample used, consisting only of university students. Furthermore, the present study attests to the discriminant validity of the LSAS-SR for the Brazilian context, supporting its use for screening for clinical or subclinical SAD cases in the clinical and research contexts.

Further studies were conducted to verify the other psychometric qualities of the LSAS-SR, i.e.: concurrent validity, divergent validity, factorial analysis, test-retest reliability and internal consistency, in clinical samples as well as in the Brazilian general population, and the results indicate the excellent psychometric properties of the scale and its suitability for use in this context [20].

## Conclusions

In summary, it can be said that the initial aims of the study were achieved, since the discriminative validity of the instrument was measured with methodological precision, and the identified cutoffs allowed the identification of the different groups studied.

The importance of cross-cultural validation studies is related to the adequacy of the evaluation parameters of each instrument for a particular sociocultural context. In Brazil, the LSAS-SR had not had its psychometric proprieties measured until now, which limited the uses of the instrument. Accordingly, in view of these results, the LSAS-SR can be considered an instrument for screening for SAD in the Brazilian context, despite the limitations of the study.

## References

[1] American Psychiatric Association: (2000). *Diagnostic and statistical manual of mental disorders* (4th ed., *text revision*). Washington, DC: 2000.

[2] Crum RM, Pratt LA: Risk of heavy drinking and alcohol use disorders in social phobia: a prospective analysis. Am J Psychiatry 2001, 158 (10):1693–700. 11579004 10.1176/appi.ajp.158.10.1693

[3] Osório FL, Crippa JAS, Loureiro SR: Instruments for the evaluation of social phobia In Social phobia: Etiology, diagnosis and treatment. Edited by Axelby CP. Hauppauge: Nova Science Publishers; 2009: 1–66.

[4] Liebowitz M. R: Social phobia. Mod Probl Pharmacopsychiatry 1987, 22(1):147–173. 10.1159/000414022 2885745

[5] Cox BJ, Ross L, Swinson RP, Derenfeld DM: A comparison of social phobia outcome measures in cognitive-behavioral group therapy. Behav Modif 1998, 22(3): 285–297. 9670801 10.1177/01454455980223004

[6] Heimberg RG, Horner KJ, Juster HR, Safren SA, Brown EJ, Schneier FR et al: Psychometric properties of the Liebowitz Social Anxiety Scale. Psychol Med 1999, 29(1): 199–212. 10077308 10.1017/s0033291798007879

[7] Bobes J, Badía X, Luque A, García M, González MP, Dal-Ré R. Validación de las versiones en español de los cuestionarios Liebowitz Social Anxiety Scale, Social Anxiety and Distres Scale y Sheehan Disability Inventory para la evaluación de la fobia social. Med Clin (Barc) 1999, 112(14):14–16. 10363239

[8] Heimberg RG, Holaway RM: Examination of the known-groups validity of the Liebowitz Social Anxiety Scale. Depress Anxiety 2007, 454(6):447–454. 10.1002/da.20277 17120297

[9] Kummer A, Cardoso F, Teixeira AL: Frequency of social phobia and psychometric properties of the Liebowitz social anxiety scale in Parkinson’s disease. Mov Disorders 2008, 23(12):1739–1743. 10.1002/mds.22221 18661550

[10] Levin JB, Marom S, Gur S, Wechter D, Hermesh H: Psychometric properties and three proposed subscales of a self-report version of the Liebowitz Social Anxiety Scale translated into Hebrew. Depress anxiety 2002, 16(4):143–151. 12497645 10.1002/da.10064

[11] Mennin DS, Fresco DM, Heimberg RG, Schneier FR, Davies SO, Liebowitz MR: Screening for social anxiety disorder in the clinical setting: using the Liebowitz Social Anxiety Scale. J Anxiety Disord 2002, 16(6):661–673. 12405524 10.1016/s0887-6185(02)00134-2

[12] Oakman J, Van Ameringen M, Mancini C, Farvolden P: A confirmatory factor analysis of a self-report version of the Liebowitz Social Anxiety Scale. J Clin Psychol 2003, 59(1):149–161. 12508338 10.1002/jclp.10124

[13] Rytwinski NK, Fresco DM, Heimberg RG, Coles ME, Liebowitz MR, Cissell S et al: Screening for social anxiety disorder with the self-report version of the Liebowitz Social Anxiety Scale. Depress Anxiety 2009, 26(1):34–38. 10.1002/da.20503 18781659

[14] Soykan Ç, Özgüven HD, Gençöz T: Liebowitz Social Anxiety Scale: The Turkish version. Psychol Rep 2003, 93(7): 1059–1069. 14765570 10.2466/pr0.2003.93.3f.1059

[15] Yao SN, Note I, Fanget F, Albuisson E, Bouvard M, Jalenques I et al,: Social anxiety in patients with social phobia: validation of the Liebowitz Social Anxiety Scale: the French version. L'Encéphale 1999, 25(5): 429–435. 10598306

[16] Baker SL, Heinrichs N, Kim H-J, Hofmann SG: The Liebowitz Social Anxiety Scale as a self-report instrument: a preliminary psychometric analysis. Behav Res Ther 2002, 40(6):701–715. 12051488 10.1016/s0005-7967(01)00060-2

[17] Fresco DM, Coles ME, Heimberg RG, Liebowitz MR, Hami S, Stein MB et al: The Liebowitz Social Anxiety Scale: a comparison of the psychometric properties of self-report and clinician-administered formats. Psychol Med 2001, 31(6):1025–1035. 11513370 10.1017/s0033291701004056

[18] Forni-Santos L, Osório FL, Loureiro SR, Hallak JE, Crippa JAS: Pharmacological treatments for social anxiety disorder: are there new parameters today? Rev Psiq Clín 2011, 38(6):238–246.

[19] Osório FL, Crippa JAS, Loureiro S.R: Instrumentos de avaliação do transtorno de ansiedade social. Rev Psiq Clín 2005, 32:73–83.

[20] Forni-Santos L., Loureiro SR, Crippa JAS, Osório FL: Psychometric validation study of the liebowitz social anxiety scale—self-reported version for Brazilian Portuguese. PLosOne 2013, 8(7): e70235 10.1371/journal.pone.0070235 23922961 PMC3724831

[21] Connor KM, Kobak KA, Churchill LE, Katzelnick D, Davidson JR: Mini-Spin: A brief screening assessment for generalized social anxiety disorder. Depress Anxiety 2001, 14(2): 137–140. 11668666 10.1002/da.1055

[22] Osório FL, Crippa JAS, Loureiro SR: A study of the discriminative validity of a screening tool (MINI-SPIN) for social anxiety disorder applied to Brazilian university students. Eur Psychiatry 2007, 22(4):239–243. 17346942 10.1016/j.eurpsy.2007.01.003

[23] Osório FL, Crippa JAS, Loureiro SR: Further study of the psychometric qualities of a brief screening tool for social phobia (MINI-SPIN) applied to clinical and nonclinical samples. Perspect psychiatr C 2010, 46(4):266–278. 10.1111/j.1744-6163.2010.00261.x 20883433

[24] First MB, Spitzer RL, Gibon M, Willians JBW: *Structured Clinical Interview for DSM-IV Axis I Disorders—Clinician version (SCID-CV)*. Washington, DC: American Psychiatric Press; 1997.

[25] Del-Ben CM, Vilela JAA, Crippa JAS, Hallak JEC, Labate CM, Zuardi AW: Test-retest reliability of the Structured Clinical Interview for DSM-IV—Clinical Version (SCID-CV) translated into Portuguese. Rev Bras Psiquiatr 2001, 23: 156–159.

[26] SPSS Incorporation: (2001). *SPSS for Windows: Statistical package for the social sciences* *Release 13.0*. Chicago: 2001.

[27] Hsiao JK, Bartko JJ, Potter WZ: Diagnosing diagnoses: Receiver operating characteristic methods and psychiatry. Arch Gen Psychiatry 1989, 46(7):664–667. 2735814 10.1001/archpsyc.1989.01810070090014

[28] Baptista CA, Loureiro SR, Osório FL, Zuardi AW, Magalhães PV, Kapczinski F et al: Social phobia in Brazilian university students: Prevalence, under-recognition and academic impairment in women. J Affect Disord 2012, 136(3): 851–861. 10.1016/j.jad.2011.09.022 22018945

[29] Chagas MHN, Nardi AE, Manfro GG, Hetem LAB, Andrada NC, Levitan MN et al: Guidelines of the Brazilian Medical Association for the diagnosis and differential diagnosis of social anxiety disorder. Rev Bras Psiquiatr 2010, 32(4): 444–452. 21308267 10.1590/s1516-44462010005000029

[30] Edelmann RJ, Asendorf J, Contarello A, Zammuner V, Georgas J, Villanueva C: Self-reported expression of embarrassment in five European cultures. J Cross Cult Psychol 1989, 20(4): 371–375.

